# Virtual body image exercises for people with obesity – results on eating behavior and body perception of the ViTraS pilot study

**DOI:** 10.1186/s12911-025-02993-x

**Published:** 2025-04-25

**Authors:** Kathrin Gemesi, Nina Döllinger, Natascha-Alexandra Weinberger, Erik Wolf, David Mal, Sebastian Keppler, Stephan Wenninger, Emily Bader, Carolin Wienrich, Claudia Luck-Sikorski, Marc Erich Latoschik, Johann Habakuk Israel, Mario Botsch, Christina Holzapfel

**Affiliations:** 1 Institute for Nutritional Medicine, School of Medicine and Health, Technical University of Munich, Munich, Germany; 2 Psychology of Intelligent Interactive Systems (PIIS) Group, University of Würzburg, Würzburg, Germany; 3 SRH, University of Applied Health Sciences, Research Group “Chronic Diseases and Psychological Health” (COPE), Gera, Germany; 4 Human-Computer Interaction (HCI) Group, University of Würzburg, Würzburg, Germany; 5 Human-Centered Immersive Systems (CENTIS) Group, HTW Berlin – University of Applied Sciences, Berlin, Germany; 6 Computer Graphics Group, Technical University of Dortmund, Dortmund, Germany; 7 Integrated Research and Treatment Center (IFB) Adiposity Diseases, Leipzig University – Medical Center, Leipzig, Germany; 8 Department of Nutritional, Food and Consumer Sciences, Fulda University of Applied Sciences, Fulda, Germany

**Keywords:** Virtual reality, Feasibility, Cognitive behavioral therapy, Mirror exposition

## Abstract

**Background:**

A negative body image can have an impact on developing and maintaining obesity. Using virtual reality (VR) to conduct cognitive behavioral therapy (CBT) is an innovative approach to treat people with obesity. This multicenter non-randomized pilot study examined the feasibility and the effect on eating behavior and body perception of a newly developed VR system to conduct body image exercises.

**Methods:**

Participants with a body mass index (BMI) ≥ 30.0 kg/m^2^ without severe mental diseases attended three study visits in an interval of one to four weeks to receive virtual (VR intervention) or traditional (non-VR intervention) body image exercises. Data on anthropometrics, eating behavior (Dutch Eating Behavior Questionnaire, DEBQ), body perception (Body Shape Questionnaire, BSQ; Multidimensional Assessment of Interoceptive Awareness, MAIA), and satisfaction (standardized interview and questionnaire) were collected.

**Results:**

In total, 66 participants (VR intervention: 31, non-VR intervention: 35) were included. The majority was female (52/66, 78.8 %), the mean age was 45.0 ± 12.8 years, and the mean BMI was 36.8 ± 4.3 kg/m^2^. Both intervention groups showed non-significant body weight reduction (VR intervention: 1.7 ± 3.3 %, non-VR intervention: 0.9 ± 3.0 %) and showed no statistically significant difference between the groups (*p* = 0.35). Scores of DEBQ, BSQ, and MAIA showed over time no statistically significant changes neither between the two groups nor within the groups (all *p* ≥ 0.05). The overall satisfaction of the VR group with the two virtual body image exercises was high (4.1 ± 0.8 on a 5-point Likert scale).

**Conclusions:**

The intervention with the developed VR system was feasible and the virtual and traditional body image exercises resulted in statistically non-significant weight loss. It seems that single focus on body image is not successful in improving eating behavior and body perception in people with obesity. Long-term human intervention studies with larger sample sizes are necessary to examine the efficacy of integrating this kind of VR system into standard obesity therapy.

**Trial registration:**

This study was registered in the German Clinical Trials Register (Registration number: DRKS00027906, Date of registration: 8^th^ February 2022).

## Introduction

Overweight and obesity have grown to a global public health challenge affecting almost 60 % of adults in the WHO European region [1]. Evidence-based guidelines recommend a comprehensive multimodal treatment delivered by an interdisciplinary team to cover the main lifestyle factors nutrition and physical activity as well as behavior [2].

According to the European Guidelines for Obesity Management in Adults, cognitive behavioral therapy (CBT) should support obesity treatment to understand patients’ thoughts and beliefs concerning weight and weight control, obesity and its consequences [3]. Specific behaviors are addressed with the help of e.g. self-monitoring, stimulus control, and cognitive and relaxation techniques. Body image is another element that is mentioned in the context of obesity therapy [2, 3] and could be identified by Teixeira et al. [4] as predictor for successful weight management. According to a systematic review and meta-analysis, persons with obesity report a higher body image dissatisfaction compared to persons with normal weight and especially women seem to be affected [5].

As guidelines for obesity therapy recommend, elements of CBT should not only be used by psychotherapists and psychiatrists [2, 3]. According to a survey among nutrition experts conducting obesity therapy, behavior treatment approaches like promotion of motivation, target agreements, and relapse prevention are part of their nutrition counseling sessions [6]. Additionally, nutritionists stated to address body image with their patients with overweight or obesity but rarely use body image therapy approaches like drawing exercises and mirror exposition [6]. This is not surprising since nutrition experts traditionally are not trained in behavioral change techniques [7]. As a main contact person for people with overweight and obesity they have to provide at least some behavioral change skills [6]. A survey among patients with overweight and obesity about the necessity and use of body image therapy as part of obesity treatment confirmed these findings [8].

Virtual reality (VR) technology, especially the use of (personalized) avatars (= virtual self-representations), could serve as a supporting and effective tool for nutrition experts to incorporate elements of CBT and body image therapy into weight loss therapy [6]. Based on current literature, avatar-based VR interventions have been shown to impact body image and/or body satisfaction [9, 10], and can even be effective for short- to medium-term weight loss (four weeks to six months) and long-term weight maintenance (12 months) in people with obesity [9]. Moreover literature suggests that personalized avatars have a positive effect on self-perception, behavior and engagement [11, 12]. According to Giuseppe Riva’s theory a “locked allocentric negative body image” caused by internal and/or external stressors may be restored by using VR interventions [13]. The allocentric (third-person) body image is shaped by emotions and beliefs, and is normally adjusted by the egocentric (first-person) body image, which is shaped by perception. Impaired neural mechanisms prevent this correction and lock the allocentric negative body image in place. As a result, even significant changes in diet or weight fail to alleviate body image dissatisfaction, sometimes resulting in a cycle of unsuccessful attempts. At this point, VR could be a tool to restore the balance of allocentric and egocentric body image by conducting exposure therapy in a controlled environment [13].

The project “Virtual Reality Therapy by Stimulation of Modulated Body Perception (ViTraS)” [14] explores the development of new technology-driven therapy approaches involving personalized avatars for people with obesity based on the Behavioral Framework of Immersive Technologies (BehaveFIT) [15]. The primary aim of the ViTraS pilot study was to evaluate in a hypothesis-free approach the feasibility and effect of virtual body image exercises delivered by a VR system including the embodiment of a personalized avatar and virtual mirror exposition in people with obesity in comparison with similarly conducted non-virtual body image exercises.

## Methods

### Study population

This multicenter pilot study was conducted at the Human-Technology-Systems department of the University of Würzburg, the SRH University of Applied Health Sciences in Gera, and the Institute for Nutritional Medicine at the School of Medicine and Health of the Technical University of Munich. The study protocol was approved by the local ethics committees (Würzburg: 8^th^ November 2021; Gera: 2021-2484-BO; Munich: 90/22 S) and was registered at the German Clinical Trials Register (Registration number: DRKS00027906). All participants gave written informed consent before participation.

Participants were recruited through social media, advertisement in newspapers, and flyers. Participants with the following inclusion criteria were included into the study: adults (women, men), aged 18 years and older, body mass index (BMI) ≥ 30.0 kg/m^2^, with stable self-reported body weight (± 5 kg) in the last three months, no obesity therapy in the last six months, and without severe mental diseases (Patient Health Questionnaire-2 score ≤ 3, not on medication with antidepressants, no psychotherapy in the last six months). Inclusion criteria were checked through a screening phone call and verified at the first on-site visit. Included participants were allocated to two intervention groups (VR and non-VR) depending on the place of residence. The VR intervention was conducted in Würzburg, where the necessary VR system was provided, and the non-VR intervention was provided in Gera and Munich. In consequence, the participants were not allocated randomly and there was no control group.

**Fig. 1:**
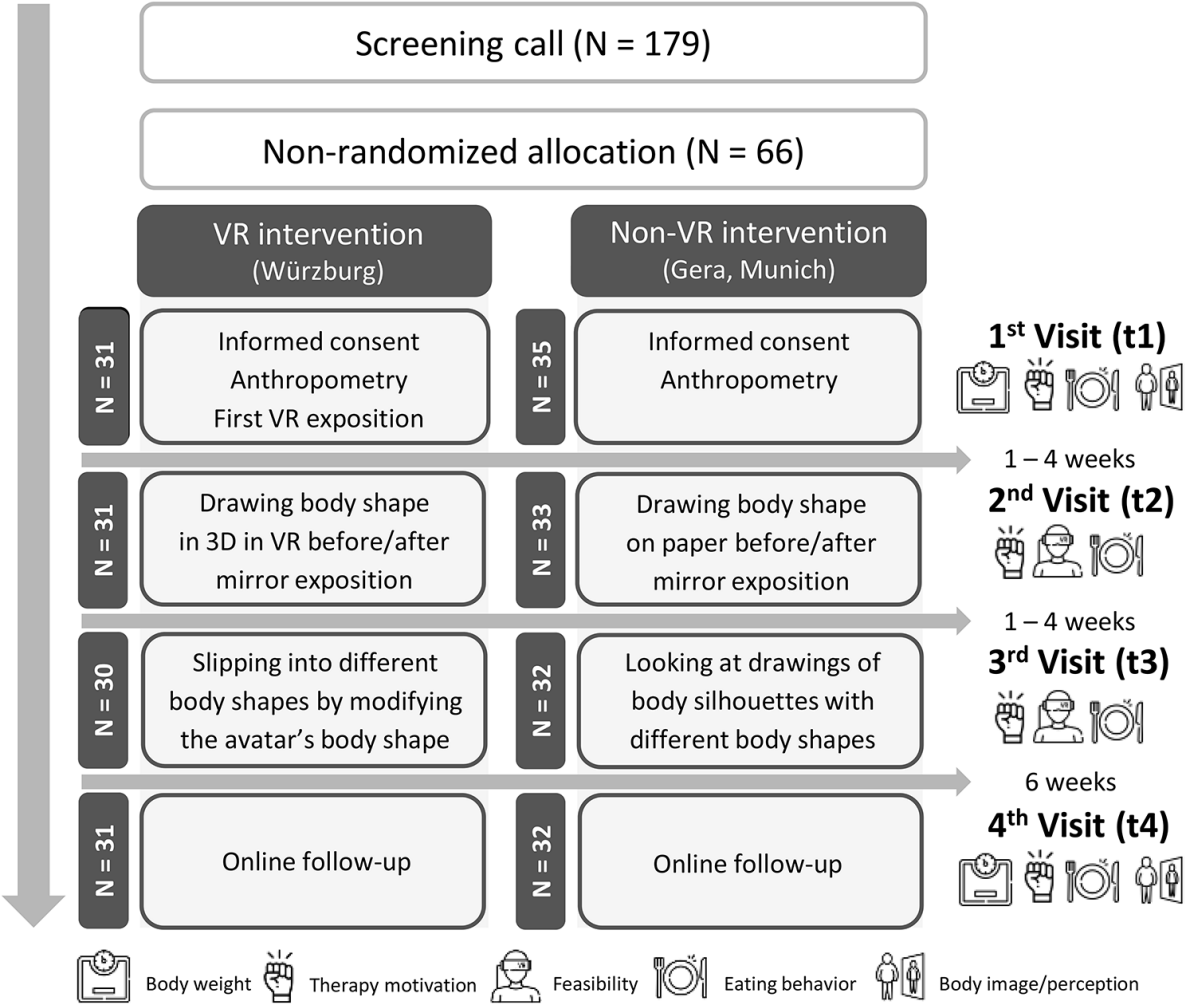
Study flow chart

Participants of both groups attended three on-site study visits (t1–t3) in an interval of one to four weeks. The first on-site study visit (t1) on-boarded the participants (written informed consent, check of inclusion criteria) and prepared them (especially the VR group) for the intervention. The two body image exercises were conducted in the following two separate study visits (t2 and t3) to minimize the duration per visit. Six weeks (t4) after the last on-site visit (t3), the participants filled out a digital follow-up questionnaire. The average study duration was 14 weeks per participant (Fig. 1).

### Intervention

A standardized interview guideline was used in both intervention groups to give instructions for exercises, to collect qualitative data in a standardized manner, and to guarantee comparable intervention processes. However, the duration of a participant’s exercise completion (in both groups) and the duration of the VR exposition in the VR group could vary among participants.

#### VR intervention

**Fig. 2:**
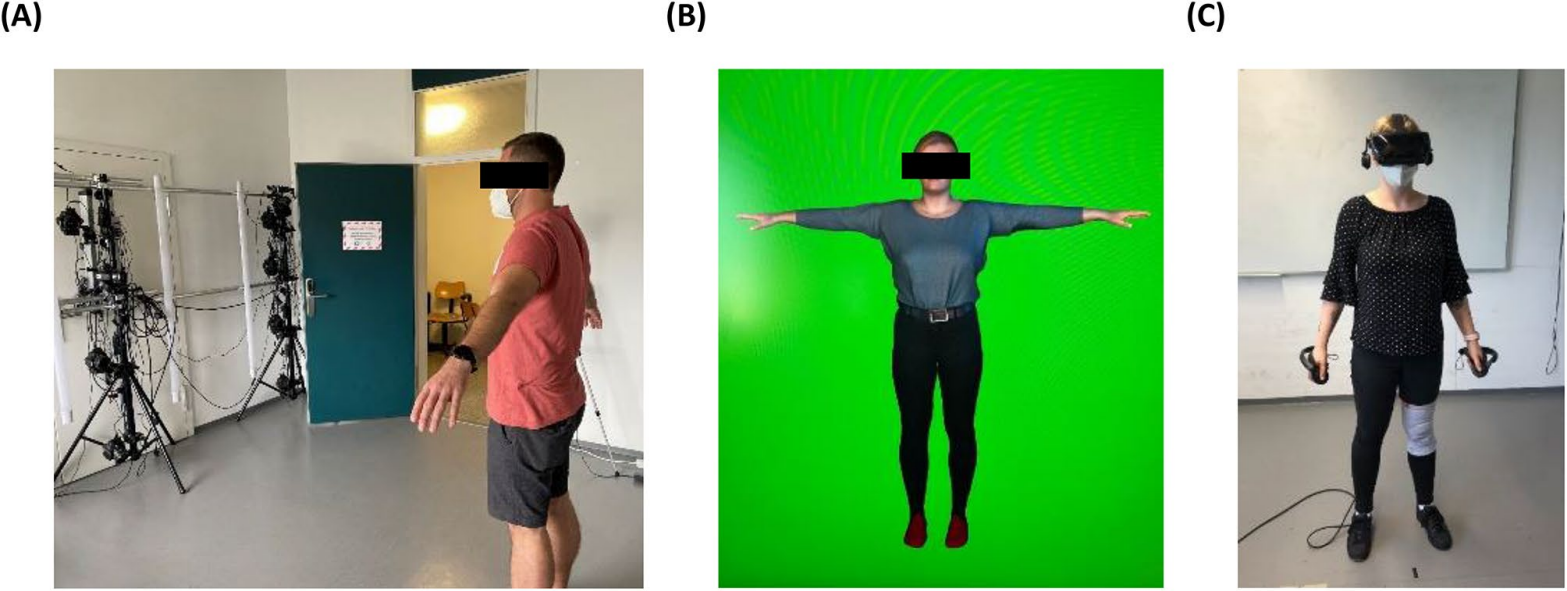
VR technology with (**A**) body scanner, (**B**) avatar, and (**C**) VR glasses and hand controllers

For the VR intervention group a personalized realistic virtual 3D model (also called “avatar”) was generated at the first visit. To this end, a body scanner (custom-made photogrammetry rig) consisting of 15 Canon EOS 2000D cameras arranged in a 5 × 3 grid was used to capture the participants’ body from four sides (Fig. 2A) [16]. To process the four partial scans, the existing avatar generation pipeline from Achenbach et al. [17] was used and adapted accordingly, yielding a fully animatable avatar of the scanned person (Fig. 2B). Participants were introduced to their avatar at the first visit, while it was used in the following two on-site visits for the body image exercises. When doing these exercises, participants embodied their avatar from a first-person perspective while observing it via a Valve Index head-mounted-display (HMD) and interacted with it using two Valve Index hand controllers (Fig. 2C). The VR system was implemented using Unity version 2020.3.18 LTS [18]. The HMD was tracked by three SteamVR Basisstations 2.0 and integrated using SteamVR version 1.21.4 (Valve Corporation) and its corresponding Unity plugin version 2.7.2 (SDK 1.14.15). The participants’ body was tracked by using eight FLIR Blackfly SBFS-PGE-1682C RGB cameras which provided input for the tracking software “Capture Live” version 248 by TheCaptury, running on a Ubuntu workstation powered by an Intel Core i7-9700K, an Nvidia RTX 2080 Ti, and 32 GB of RAM, streaming to TheCaptury’s corresponding Unity plugin [18]. A more detailed description of the whole VR system used can be found elsewhere [18, 19]. In the range of healthy participants in other studies, the embodiment was in an upper, very good range on the sub-dimensions of agency and ownership [20].

**Fig. 3:**
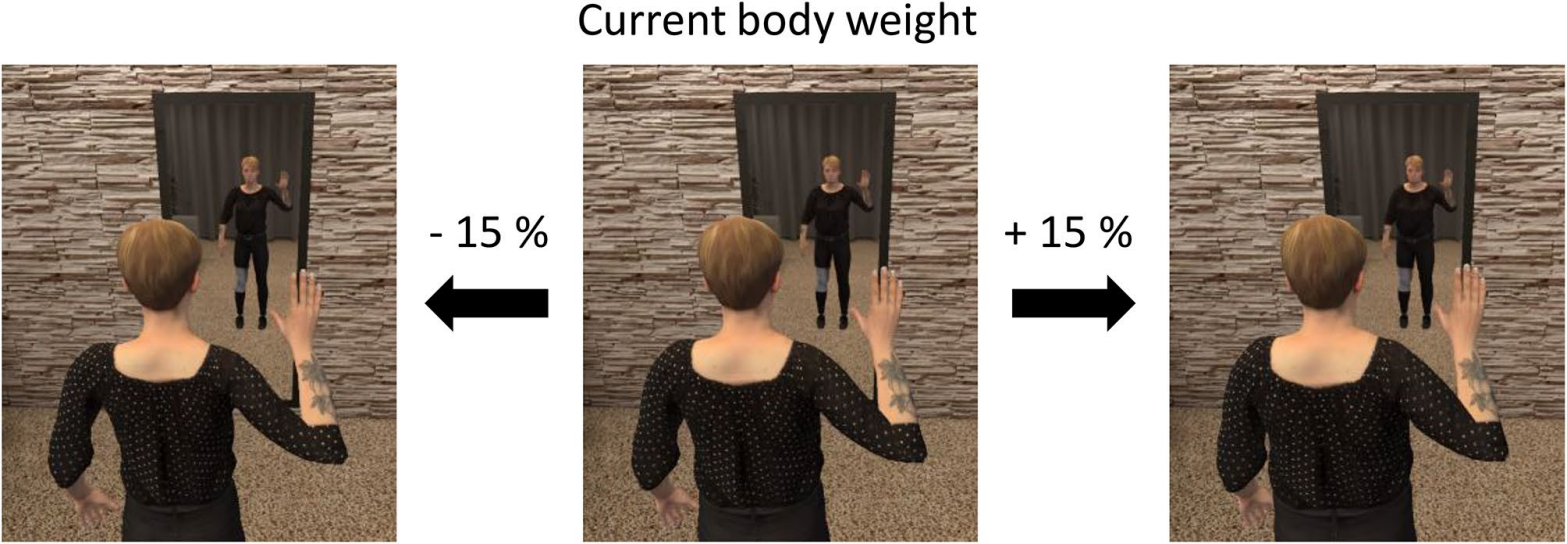
Virtual mirror exposition and avatar body shape modification

At the second visit, participants of the VR intervention group performed the first virtual body image exercise which included drawing their own current body shape in real size using a virtual pen (Logitech VR Ink Pilot-Edition) before and after looking into a virtual full-length mirror (= virtual mirror exposition, according to Hilbert and Tuschen-Caffier [21]) [22, 23]. At the third visit, participants could modify the avatars’ body shape (Fig. 3) to visualize what body shape they would be happy with in six or twelve months, and what their desired body shape would look like. To allow participants to change the avatar’s body shape, a statistical body weight modification approach was used [18].

#### Non-VR intervention

The non-VR intervention group received the same body image exercises in a traditional way based on existing manuals. At the second visit, participants of the non-VR group had to do the drawing exercise on a big sheet of paper (DIN A0) with a pencil according to Legenbauer and Vocks [24] and Munsch et al. [25] before and after looking into a full-length mirror (mirror exposition according to Hilbert and Tuschen-Caffier [21]). On the third visit, participants of the non-VR group received a series of body silhouettes (low to high body mass, women and men) by Lønnebotn et al. [26] to pick the body shape they would be happy with in six or twelve months, and their desired body shape.

### Data collection and processing

Sociodemographic and health data (e.g. weight history, diet) was collected at the first visit (t1) through a standardized questionnaire. Body weight and height were measured at the first study visit by using a stadiometer (Würzburg: Soehnle 5003, Soehnle Industrial Solutions GmbH, Germany; Gera: simple measuring stick at the wall; Munich: SECA 214, Seca GmbH & Co. KG, Germany) and a bio impedance analysis scale (Würzburg: Tanita MC 780MA, Tanita Europe B. V., Netherlands; Munich: Tanita BC-418MA, Tanita Europe B. V., Netherlands) or a digital personal scale (Gera: Seca GmbH & Co. KG, Germany). At the last visit (t4), self-reported data on body weight was collected by an online questionnaire. The BMI has been calculated as body weight (in kg) divided by the square of body height (in m).

For a fast assessment of therapy motivation for weight reduction, a self-developed standardized questionnaire was provided at all study visits. Participants were asked “Are you currently motivated to strive for weight loss?” with the answer options “yes” or “no”. If yes, they were further asked about their desired amount of weight reduction (“If yes: by how many kilograms?”) and about their preferred method of weight reduction (“If yes: how do you want to reduce your body weight?” → answer options: nutrition therapy, physical therapy, psychotherapy, other).

The feasibility of the VR system was evaluated at the end of the second and third visit. Therefore, a standardized questionnaire including the question “How satisfied are you with today’s exercise in [virtual body drawing or virtual body modification]?” was used. Participants of the VR group were asked to rate their satisfaction on a 5-point Likert scale ranging from “not satisfied at all” (=1) to “very satisfied” (= 5). Additionally, the participants were asked the question “How did you experience today’s exercise?” and the study team transcribed the participants’ verbal responses.

To assess eating behavior, the validated (Cronbach’s alpha = 0.89, 0.92, and 0.94) German version of the Dutch Eating Behavior Questionnaire (DEBQ) [27, 28] was used. Three domains (= eating styles) are covered by 30 items (restraint: 10 items, emotional eating: 10 items, external eating: 10 items). Participants could answer on a five-point Likert scale (1 = “never” to 5 = “very often”). Per domain, a mean score was calculated with a higher mean score indicating a greater configuration of the prevailing eating style.

To collect data on body shape concerns at baseline (t1) and at follow-up (t4) a validated short form of the German version of the Body Shape Questionnaire (BSQ) [29] with 16 items (Cronbach’s alpha > 0.90 [30]) was used. Answers were given on a six-point rating scale (1 = “never” to 6 = “always”). A mean sum score was calculated with a higher score indicating a greater concern with the body shape.

With the validated 37-item Multidimensional Assessment of Interoceptive Awareness Version 2 (MAIA-2) questionnaires (Cronbach’s alpha between 0.74 and 0.83) [31], data on participants’ everyday life body awareness was collected at baseline (t1) and at follow-up (t4). A set of three to seven items representing one of the eight subscales “noticing”, “non-distracting”, “not worrying”, “attention regulation”, “emotional awareness”, “self-regulation”, “body listening”, and “trusting” was calculated. A higher mean score indicates a greater tendency toward the prevailing behavior. In the following, body shape concerns and body awareness will be summarized as “body perception”.

All questionnaires were provided to the study participants digitally through the platform SoSci Survey.

### Statistical analysis

Integrity and plausibility checks were performed. Due to incomplete participation, varying sample sizes resulted per visit. Additionally, any implausible responses from the interviews were excluded from the analysis.

For descriptive analysis, absolute and relative frequency, means, and standard deviations (SD) were calculated. Two-sample t-test (for normally distributed outcomes) or a Mann-Whitney-U test (for non-normally distributed outcomes) were used 1) to compare baseline characteristics and data on obesity therapy history between the groups and 2) to compare the satisfaction of the VR group after the two virtual body image exercises. Normality was tested by using the Shapiro-Wilk test and by graphical inspection of the distribution in each group. Variance homogeneity was checked by using Levene’s test. For categorical outcomes, Fisher’s exact test or Pearson’s chi-squared test was used. Multiple linear regression analysis adjusted for gender, age, and baseline body weight was conducted for group comparison of changes in body weight, therapy motivation, eating behavior, and body perception. P-values <0.05 were considered as statistically significant. All analyses were performed using RStudio (V4.1.0).

## Results

### Baseline data

After assessing for eligibility, 66 participants were included into the study between 28^th^ March 2022 (first patient in) and 20^th^ February 2023 (last patient out). In total, 31 persons were allocated to the VR intervention group and 35 to the non-VR intervention group (Fig. 1). All participants of the VR group completed the last visit. However, one participant had to stop the body image exercise at t3 because the person had an emotive reaction. In the non-VR group, three participants did not complete the last visit resulting in a dropout rate of 8.6 % (3/35). One participant stopped answering the study team after the baseline visit (t1) and two participants could not keep the interval of maximum four weeks between the study visits. The total dropout rate is 4.5 % (3/66).

As shown in Table 1, 78.8 % (52/66) of the participants were female, the mean age was 45.0 ± 12.8 years, and the mean BMI was 36.8 ± 4.3 kg/m^2^. In total, most of the participants were married (32/66, 48.5 %) and highly educated (41/66, 62.1 %). About one quarter of the population had hypertension (19/66, 28.8 %) or/and thyroid disease (17/66, 25.8 %). Furthermore, 42.2 % quoted to follow a balanced diet (Table 1). In summary, the two groups did not differ significantly at baseline (all *p* ≥ 0.05).

**Table 1:** Baseline characteristics of total participants (N = 66)

	VR (N = 31)	Non-VR (N = 35)	*P*-value
	n (%) or mean ± SD	n (%) or mean ± SD	
**Gender**^a^			0.58
Female	23 (74.2)	29 (82.9)	
Male	8 (25.8)	6 (17.1)	
**Age (years)**^b^	41.9 ± 12.0	47.8 ± 13.1	0.06
**Body weight (kg)**^c^	104.9 ± 17.9	105.7 ± 16.8	0.72
**Body height (m)**^b^	1.7 ± 0.1	1.7 ± 0.1	0.93
**BMI (kg/m**^**2**^**)**^a,c^	36.7 ± 4.5	36.9 ± 4.2	0.74
30.0–34.9	12 (38.7)	13 (37.1)	0.95
35.0–39.9	13 (41.9)	16 (45.7)	
≥40.0	6 (19.4)	6 (17.1)	
**Marital status**^d^			0.96
Single	13 (41.9)	15 (42.9)	
Married	15 (48.4)	17 (48.6)	
Divorced	3 (9.7)	2 (5.7)	
Widowed	0	1 (2.9)	
**Education (years)**^d^			0.88
8/9	3 (9.7)	3 (8.6)	
10	9 (29.0)	10 (28.6)	
12/13	19 (61.3)	22 (62.9)	
**Comorbidities**^d^			
Hypertension	7 (22.6)	12 (34.3)	0.41
Thyroid disease	9 (29.0)	8 (22.9)	0.59
Diabetes mellitus	4 (12.9)	5 (14.3)	1
Allergy/food intolerance	5 (16.1)	4 (11.4)	0.72
Eating disorder	3 (9.7)	3 (8.6)	1
Depression (affective disorder)	3 (9.7)	3 (8.6)	1
Dyslipidemia	1 (3.2)	5 (14.3)	0.20
Body schema disturbance	2 (6.5)	3 (8.6)	1
Anxiety disorder	0	2 (5.7)	0.49
Other	7 (22.6)	8 (22.9)	0.77
**Diet**^d^			0.85
Balanced diet	12 (38.7)	16 (45.7)	
Low carb	5 (16.1)	3 (8.6)	
Protein rich	2 (6.5)	4 (11.4)	
Low fat	2 (6.5)	3 (8.6)	
Vegetarian/vegan	2 (6.5)	1 (2.9)	
Other	8 (25.8)	8 (22.9)	

^a^Categorical variable, Pearson’s chi-squared test used for comparison

^b^Normally distributed, Variance homogeneity, Two sample t-test used for comparison

^c^Not normalliy distributed, Mann-Whitney-U test used for comparison

^d^Categorical variable, Fisher’s exact test used for comparison

Participants of the two intervention groups did not differ significantly regarding their weight loss history (Table 2). Participants reported that their highest body weight reduction was 18.4 ± 10.7 kg in the VR group and 14.3 ± 11.0 kg in the non-VR group (*p* = 0.09). According to participants’ estimation, 51.7 % (31/60) tried less than ten times to reduce their body weight in their lifetime and 48.3 % (29/60) tried between ten and fifty times (*data not shown*).

**Table 2:** Obesity therapy history of total participants (N = 66)

	VR (N = 31)	Non-VR (N = 35)	P-value
	n (%) or mean ± SD	n (%) or mean ± SD	
**Highest amount of weight loss (kg)**^a,b^	18.4 ± 10.7	14.3 ± 11.0	0.09
**Highest body weight (kg)**^b,c^	111.4 ± 18.0	111.6 ± 19.1	0.85
**Weight loss methods used (n)**^b,d,e^	5.3 ± 2.0	5.2 ± 2.1	0.81
Eating more vegetables and fruits	27 (87.1)	28 (80.0)	0.52
Increasing physical activity	26 (83.9)	29 (82.9)	1
Counting calories	26 (83.9)	25 (71.4)	0.26
Drinking more water	22 (71.0)	21 (60.0)	0.44
Program/course	17 (54.8)	19 (54.3)	1
App	15 (48.4)	15 (42.9)	0.80
Together with a friend	11 (35.5)	13 (37.1)	1
Professional support	7 (22.6)	11 (31.4)	0.58
Asking a physician for advice	8 (25.8)	9 (25.7)	1
Other	4 (12.9)	11 (31.4)	0.09

^a^Question: “Your biggest weight loss to date was?”

^b^Not normally distributed, Mann-Whitney-U test used for comparison

^c^Question: “What was your highest body weight?”

^d^Question: “What method(s) have you used to lose weight?”, multiple answers possible

^e^Categorical variable, Fisher’s exact test used for comparison

### Feasibility

The VR intervention group reported an average satisfaction of 4.0 ± 0.9 after the first virtual body image exercise (t2) and 4.3 ± 0.5 after the second virtual body image exercise (t3), with no statistically significant difference between the two study visits (*p* = 0.22, *data not shown*). The overall satisfaction for both body image exercises together was 4.1 ± 0.8 (*data not shown*).

Regarding the body image exercise experience at t2, participants in the VR group positively noted that the drawing exercise was interesting, fun, and gave them a good feeling during and after the exercise. Some participants negatively noted the handling of the virtual pen, the 3D drawing, the shape of their avatar, and the room where the exercise was carried out. Four study participants recommended that virtual drawing in 2D and more practice time beforehand would enhance the exercise.

Regarding the body image exercise at t3, participants in the VR group positively noted that the virtual body modification was interesting, fun, motivating, quite realistic, and easier and more enjoyable to do than the virtual drawing exercise. The avatar’s shape and the unrealistic changes to the avatar when making it thinner, including excessive or insufficient shrinking of shoulder width and the remaining skin folds, were noted negatively. Six participants recommended making the experience more realistic by enabling the separate modification of body parts. One participant suggested that a direct comparison between the modified avatar and the original avatar would be better than presenting them separately.

### Changes of body weight

After six weeks of follow-up, a non-significant weight reduction of 1.6 ± 3.0 kg (1.7 ± 3.3 %) in the VR group and 0.9 ± 3.4 kg (0.9 ± 3.0 %) in the non-VR group without a significant difference between the groups (β [95 % CI] = ‒0.8 kg [‒2.5; 0.9], *p* = 0.35, effect size = 0.13, *data not shown*) was found.

### Changes of therapy motivation

In Table 3, data about therapy motivation at four time points are shown. All participants answered at all study visits on the question “Are you currently satisfied with your body weight?” with “Yes” (*data not shown*). At all three on-site visits, participants stated in total to desire to loose approximately 20 % of their current body weight. After six weeks of follow-up (t4), participants desired weight reduction was 18.4 ± 9.5 % (Table 3). No statistically significant differences between VR and non-VR group could be shown (all *p* ≥ 0.05, Table 3). Examining changes within the groups, a statistically significant difference within the VR group was found (Friedman rank sum test: *p* = 0.006, Post-hoc analysis: all *p* ≥ 0.05, *data not shown*) but not within the non-VR group (Friedman rank sum test: *p* = 0.21, *data not shown*). The most frequently selected methods, which people stated to achieve future weight loss with, were nutrition therapy and physical therapy (chosen by more than 45 % at all four time points). After the second body image exercise (t3) significantly more participants of the non-VR group selected psychotherapy as method to achieve weight loss compared to the VR group (38.7 % vs. 7.7 %, *p* = 0.01).

**Table 3:** Therapy motivation of participants from t1 to t4

	Total mean ± SD or n (%)	VR mean ± SD or n (%)	Non-VR mean ± SD or n (%)	Mean difference [95 CI]^e^	Unadjusted *P*-value	Effect size
**Motivated to lose weight**^a,b^						
t1	63/66 (95.5)	30/31 (96.8)	33/35 (94.3)	–	1	0.06
t2	56/63 (88.9)	28/30 (93.3)	28/33 (84.8)	–	0.43	0.13
t3	57/61 (93.4)	26/29 (89.7)	31/32 (96.9)	–	0.34	0.15
t4	56/62 (90.3)	27/30 (90.0)	29/32 (90.6)	–	1	0.01
**Desired weight loss (%)**^c^						
t1	20.0 ± 9.5	20.1 ± 8.4	19.8 ± 10.6	−1.7 [− 5.8; 2.4]	0.41	0.09
t2	20.0 ± 9.4	20.6 ± 8.1	19.4 ± 10.6	−1.6 [− 6.0; 2.9]	0.48	0.08
t3	20.7 ± 9.7	21.8 ± 8.5	19.7 ± 10.7	−0.4 [− 4.8; 4.0]	0.86	0.02
t4	18.4 ± 9.5	18.9 ± 8.6	17.9 ± 10.4	−2.0 [− 6.5; 2.4]	0.37	0.11
**Method**^b,d^						
t1	N = 63	N = 30	N = 33			
Nutrition therapy	39 (61.9)	14 (46.7)	25 (75.8)	–	0.02	0.30
Physical therapy	39 (61.9)	17 (56.7)	22 (66.7)	–	0.45	0.10
Psychotherapy	13 (20.6)	3 (10.0)	10 (30.3)	–	0.06	0.25
Other	14 (22.2)	7 (23.3)	7 (21.2)	–	1	0.02
t2	N = 56	N = 28	N = 28			
Nutrition therapy	33 (58.9)	13 (46.4)	20 (71.4)	–	0.10	0.25
Physical therapy	37 (66.1)	16 (57.1)	21 (75.0)	–	0.26	0.19
Psychotherapy	15 (26.8)	5 (17.9)	10 (35.7)	–	0.23	0.20
Other	18 (32.1)	10 (35.7)	8 (28.6)	–	0.78	0.08
t3	N = 57	N = 26	N = 31			
Nutrition therapy	36 (63.2)	13 (50.0)	23 (74.2)	–	0.10	0.25
Physical therapy	37 (64.9)	16 (61.5)	21 (67.7)	–	0.78	0.06
Psychotherapy	14 (24.6)	2 (7.7)	12 (38.7)	–	0.01	0.36
Other	20 (35.1)	10 (38.5)	10 (32.3)	–	0.78	0.06
t4	N = 56	N = 27	N = 29			
Nutrition therapy	34 (60.7)	14 (51.9)	20 (69.0)	–	0.27	0.18
Physical therapy	35 (62.5)	15 (55.6)	20 (69.0)	–	0.41	0.14
Psychotherapy	15 (26.8)	5 (18.5)	10 (34.5)	–	0.23	0.18
Other	18 (32.1)	11 (40.7)	7 (24.1)	–	0.25	0.18

^a^Question: “Are you currently motivated to strive for weight loss?” → yes/no

^b^Categorical variable, Fisher’s exact test used for comparison, Cramer’s V as measure for association (effect size)

^c^Question: “If yes: by how many kilograms?” (Only answered by participants who stated to be motivated to strive for weight loss.)

^d^Question: “If yes: how do you want to reduce your body weight?” → multiple answers possible (Only answered by participants who stated to be motivated to strive for weight loss.)

^e^Results are presented as unstandardized regression coefficients adjusted for gender, age, and baseline BMI (Effect size = standardized regression coefficients)

The most frequently selected reasons speaking against weight reduction were at baseline (t1) “lack of time” (VR: 38.7 %, non-VR: 37.1 %) and “low motivation” (VR: 19.4 %, non-VR: 42.9 %). After the first body image exercise (t2), participants selected “lack of time” (VR: 40.0 %, non-VR: 36.4 %) and “financial aspects” (VR: 33.3 %, non-VR: 21.2 %). After the second body image exercise (t3) and after the follow-up (t4) mainly “lack of time” (VR: 41.4 % and 50.0 %, non-VR: 28.1 % and 40.6 %) was chosen as reason against weight reduction (*data not shown*).

### Changes of eating behavior

Multiple linear regression models (adjusted for gender, age, and baseline BMI) showed that participants of the two groups did not differ significantly at any time point in their eating styles according to DEBQ scores (Table 4, all *p* ≥ 0.05). Similarly, there were no significant differences within the groups (all *p* ≥ 0.05, *data not shown*).

**Table 4:** DEBQ scores from t1 to t4 for total, VR, and non-VR group

	Total^a^	VR^b^	Non-VR^c^	**Mean difference [95% CI]**^d^	**Unadjusted P-value**	Effect size^e^
	**mean ± SD**	mean ± SD	mean ± SD			
**Restraint**						
t1	2.8 ± 0.7	2.8 ± 0.8	2.7 ± 0.7	0.1 [− 0.3; 0.5]	0.61	0.06
t2	2.8 ± 0.8	2.9 ± 0.8	2.8 ± 0.8	0.2 [− 0.3; 0.6]	0.45	0.11
t3	2.9 ± 0.9	2.9 ± 0.9	2.8 ± 0.8	0.2 [− 0.3; 0.7]	0.43	0.11
t4	3.0 ± 0.8	3.1 ± 0.8	2.8 ± 0.9	0.3 [− 0.1; 0.8]	0.16	0.20
**Emotional**						
t1	2.8 ± 1.0	2.7 ± 0.9	2.9 ± 1.0	−0.2 [− 0.7; 0.3]	0.37	0.12
t2	2.8 ± 1.0	2.7 ± 1.0	2.9 ± 1.0	−0.2 [− 0.7; 0.3]	0.37	0.12
t3	2.7 ± 1.1	2.6 ± 1.1	2.8 ± 1.1	−0.3 [− 0.8; 0.3]	0.37	0.12
t4	2.8 ± 1.0	2.7 ± 0.9	2.9 ± 1.0	−0.2 [− 0.7; 0.3]	0.39	0.12
**External**						
t1	3.4 ± 0.7	3.3 ± 0.6	3.4 ± 0.8	−0.1 [− 0.4; 0.3]	0.65	0.06
t2	3.4 ± 0.7	3.4 ± 0.6	3.3 ± 0.7	0.001 [− 0.4; 0.4]	> 0.99	0.001
t3	3.3 ± 0.8	3.4 ± 0.6	3.1 ± 0.8	0.2 [− 0.2; 0.6]	0.27	0.15
t4	3.3 ± 0.8	3.4 ± 0.7	3.2 ± 0.8	0.2 [− 0.3; 0.6]	0.43	0.11

^a^Total sample size: 64 (t1), 62 (t2), 60 (t3), 61 (t4)

^b^VR group sample size: 30 (t1), 29 (t2), 29 (t3), 29 (t4)

^c^Non-VR group sample size: 34 (t1), 33 (t2), 31 (t3), 32 (t4)

^d^Results are presented as unstandardized regression coefficients adjusted for gender, age, and baseline BMI

^e^Results are presented as standardized regression coefficients

### Changes of body perception

For BSQ and MAIA scores (Table 5), multiple linear regression models (adjusted for gender, age, and baseline BMI) showed that participants of the two groups did not differ significantly at any time point (all p ≥ 0.05) except for the subscale “not worrying” at t1 (β [95 % CI] = 0.3 [0.1; 0.5], p = 0.01). Within the groups, no statistically significant changes over time could be observed (all p ≥ 0.05, data not shown).

**Table 5:** BSQ and MAIA scores at t1 and t4 for total, VR, and non-VR group

	Total^a^	VR^b^	Non-VR^c^	**Mean difference [95% CI]**^d^	**Unadjusted P-value**	Effect size^e^
	**mean** ± **SD**	**mean** ± **SD**	**mean** ± **SD**			
**BSQ**						
t1	50.1 ± 14.7	48.3 ± 13.0	51.6 ± 16.1	−3.9 [− 10.9; 3.1]	0.27	0.13
t4	48.7 ± 15.6	49.0 ± 15.6	48.5 ± 15.7	−1.6 [− 9.6; 6.4]	0.69	0.05
**MAIA**						
Attention regulation						
t1	2.7 ± 0.9	2.8 ± 0.8	2.5 ± 0.9	0.4 [− 0.1; 0.8]	0.10	0.21
t4	2.7 ± 0.9	2.8 ± 0.7	2.7 ± 0.9	0.3 [− 0.1; 0.7]	0.17	0.18
Body listening						
t1	1.8 ± 0.8	1.8 ± 0.7	1.8 ± 0.9	0.1 [− 0.3; 0.5]	0.60	0.07
t4	1.9 ± 0.8	2.0 ± 0.8	1.8 ± 0.8	0.3 [− 0.1; 0.8]	0.14	0.21
Emotional awareness						
t1	3.5 ± 1.0	3.5 ± 1.0	3.5 ± 1.0	0.1 [− 0.4; 0.6]	0.68	0.05
t4	3.5 ± 0.9	3.6 ± 0.9	3.5 ± 0.9	0.2 [− 0.3; 0.7]	0.37	0.12
Not distracting						
t1	2.9 ± 0.9	2.9 ± 1.0	2.9 ± 0.8	−0.1 [− 0.6; 0.3]	0.57	0.07
t4	3.0 ± 0.8	2.9 ± 0.9	3.1 ± 0.7	−0.4 [− 0.9; 0.02]	0.06	0.27
Noticing						
t1	3.3 ± 0.8	3.4 ± 0.8	3.3 ± 0.8	0.3 [− 0.02; 0.7]	0.07	0.21
t4	3.3 ± 0.8	3.3 ± 0.8	3.2 ± 0.8	0.3 [− 0.2; 0.7]	0.21	0.17
Not worrying						
t1	3.1 ± 0.5	3.3 ± 0.3	2.9 ± 0.5	0.3 [0.1; 0.5]	0.01	0.33
t4	3.1 ± 0.4	3.1 ± 0.4	3.1 ± 0.4	−0.02 [− 0.2; 0.2]	0.83	0.03
Self-regulation						
t1	2.4 ± 1.1	2.4 ± 1.1	2.4 ± 1.1	0.2 [− 0.4; 0.7]	0.56	0.07
t4	2.4 ± 1.0	2.4 ± 0.9	2.5 ± 1.1	−0.002 [− 0.6; 0.6]	0.99	0.001
Trusting						
t1	2.9 ± 1.2	3.1 ± 1.2	2.8 ± 1.2	0.4 [− 0.2; 1.0]	0.19	0.17
t4	2.8 ± 1.2	3.0 ± 1.1	2.6 ± 1.3	0.5 [− 0.1; 1.2]	0.12	0.22

^a^Total sample size: BSQ → 66 (t1), 62 (t4); MAIA → 66 (t1), 61 (t4)

^b^VR group sample size: BSQ → 31 (t1), 30 (t4); MAIA → 31 (t1), 30 (t4)

^c^Non-VR group sample size: BSQ → 35 (t1), 32 (t4); MAIA → 35 (t1), 31 (t4)

^d^Results are presented as unstandardized regression coefficients adjusted for gender, age, and baseline BMI

^e^Results are presented as standardized regression coefficients

## Discussion

As part of the ViTraS pilot study, adults with obesity were given body image exercises either delivered virtually through a VR system that included a personalized avatar and a virtual mirror or non-virtually through traditional paper-pencil exercises and a real mirror. In summary, feasibility has been shown, but no significant changes could be found in the investigated parameters, such as eating behavior and body perception (including body shape concerns and body awareness), either between or within the groups.

Weight loss approaches used by study participants in the past were mainly without professional support. The minority of participants stated to want to reduce body weight with psychotherapy. These results go hand in hand with the finding that “lack of time”, “therapy availability”, and “financial issues” are the main factors keeping the participants from weight reduction. A new obesity therapy approach like the VR system developed in the ViTraS project could help overcoming these issues [6]. Firstly, it would enlarge the range of digital offers for obesity therapy and their integration would probably result in improved quality, accessibility, and long-term support [6, 32]. Nutrition experts, one of the main contact persons for people with obesity who want to reduce weight, could use this technology to provide body image exercises that are currently missing in their obesity therapy sessions [6]. Secondly, the unique ability of VR technology to visualize theoretical body shape changes by using avatars has the potential to help people with obesity to set more realistic weight loss goals [9].

The primary goal of this pilot study was to demonstrate the feasibility of a newly developed VR system that addresses body image issues in people with obesity. To achieve this, the intervention focused solely on single body image exercises and did not integrate them into a standardized weight loss program. In contrast, the study by Phelan et al. [33] examined the feasibility of a VR-enhanced behavioral weight loss program. Our simplified approach facilitated the handling of technology by both study team and study participants and the feasibility was not affected by the general elements of a weight loss program. This pilot study provided both qualitative data on feasibility (as presented) and a variety of quantitative data (*data not shown*). For instance, system performance and stability was constantly monitored by a study team member during the study visits. We used validated questionnaires to measure presence in VR (Igroup Presence Questionnaire [34]), embodiment (Virtual Embodiment Questionnaire [35]), and simulation sickness (Fast Motion Sickness Scale [36]). The compatibility of the VR system with hardware and software also supports its feasibility. The qualitative data collected through interviews showed that study participants provided positive feedback and demonstrated high user acceptance. The negative feedback was mainly related to the complexity of using the controller for the virtual drawing and the shape of the avatar, both before and after modification. In summary, the newly developed VR system is feasible, but further improvements are necessary to optimize the VR experience.

As a second objective, the effect of single body image exercises on obesity-related parameters, such as eating behavior, was examined. Carraca et al. [37] demonstrated changes in eating behavior after conducting a 12-month behavioral weight management program including body image exercises in 239 women with overweight. Eating behavior was assessed by using validated questionnaires. After 12 months, a significant effect on eating behavior change was found fully mediated by body image (effect ratio: 0.68), and eating behavior could be positively predicted by body image change (*p* < 0.001) [37]. In the ViTraS pilot study the VR system was not integrated in a weight loss program, which might be the reason, that participants did not change eating behavior. Tambone et al. [38] investigated in their study how the presentation of an avatar with a thinner or thicker body shape than one’s own body affects people’s food intake. Participants presented with a thinner avatar showed higher rejection towards high-caloric food products than before the intervention (*p* = 0.04) [38]. Similar findings could be made in a study by Kuo et al. [39] where students interested in weight reduction were presented with a personalized avatar with either their current personal body weight or a reduced body weight. Participants who were shown an avatar with reduced weight, reacted in a following test with eating less ice cream (*p* = 0.007) and were more likely to choose a sugar-free beverage as reward (*p* = 0.005) compared to the control group, which were presented with an avatar of the current body weight [39]. The two mentioned studies show that with an appropriate study design, the interaction with an avatars body shape can have an effect on food intake. In contrast, the ViTraS pilot study focused on the expression of eating styles by the validated DEBQ questionnaire rather than examining the reaction to exposed food. Given that the measured eating styles are based on psychological theories that explain the development and maintenance of obesity, it might be necessary to extend the intervention period to detect changes over time.

In addition, there was no significant change in body perception, measured by the two validated questionnaires BSQ and MAIA, in the ViTraS pilot study. In the 12-week randomized controlled trial (RCT) by Cárdenas-López et al. [40], 24 adults with obesity were randomized to the waiting-list group, to the CBT group or to the group receiving experiential cognitive therapy (ECT) in a web-based VR environment presenting critical situations for people with obesity and body image issues. According to the administered BSQ questionnaire with 34 items, participants of both treatment groups could significantly improve body shape concerns, but the ECT group more than the CBT group [40]. The MAIA questionnaire has not been widely used in individuals with obesity, however, research has shown a relationship between body awareness and obesity [41]. Compared to the validation study [31], our participants score lower in “body listening”, higher in “not distracting”, higher in “not worrying”, lower in “self-regulation”, and lower in “trusting”. To change body awareness over time, it might be necessary to conduct longer and more intensive interventions. However, irrespective of the study duration and intervention intensity, it is crucial to measure body awareness in settings including virtual self-representation by an avatar and mirror exposition [42].

To the best of our knowledge, there are currently no RCT examining the use of avatar embodiment and virtual mirror exposition to conduct body image exercises in people with obesity. Therefore, larger human intervention studies with longer intervention and follow-up periods, as well as appropriate control groups, are needed.

Manzoni et al. [43] and Thomas et al. [44] examined web-based VR-enhanced CBT within the scope of multimodal weight management programs over six months. VR-enhanced CBT addressing nutrition and behavior management was conducted in virtual environments representing critical daily situations (e.g. home, supermarket, etc.). In the study by Manzoni and colleagues [43], after six months, each group significantly lost weight without differences between the groups. After one year, significantly more participants who received the standard behavior inpatient program (SBP) plus VR-enhanced CBT maintained or improved weight loss compared to participants only receiving SBP (*p* = 0.004) [43]. Similarly, in the study by Thomas et al. [44], participants who had access to an online weight management program plus VR-enhanced CBT showed significantly greater weight loss after six months compared to the group without VR sessions (*p* = 0.042). The efficacy of the immersive VR platform “ConVRself” in adults with obesity has been investigated within the European-funded SOCRATES project [45]. Study participants either received treatment as usual (control group, CG), or used ConVRself plus received training in motivational interviewing (experimental group 1, EG1) or used only ConVRself (experimental group 2, EG2). The EG1 group was able to conduct self-talk by alternating between the embodiment of an avatar presenting themselves and an avatar of a counselor. In contrast, the EG2 group could only embody their own avatar. Both experimental groups showed significant improvements compared to the CG e.g. in confidence to lose weight (both: *p* = 0.02), readiness to exercise more (EG1: *p* = 0.03), uncontrolled eating (EG1: *p* = 0.01), emotional eating (EG1: *p* = 0.03), and anxiety (EG1: *p* = 0.01) [45].

The validity of the ViTraS pilot study is limited by the fact that 80 % of study participants were female, which is common for studies examining obesity or body image. The study design was appropriate for investigation of the feasibility of the VR technology. For examining the effect of the body image exercises conducted virtually or non-virtually, the sample size was too small. Another limitation of the study is that study participants were allocated to the two intervention groups based on place of residence, therefore, not randomized, and a control group was missing. Nevertheless, this study represents a mandatory step for further development of the VR system. Furthermore, this study is strengthened by its simple study design, which focuses on the feasibility of the VR technique.

## Conclusion

The ViTraS pilot study demonstrated the feasibility of the developed VR system. However, as a single intervention approach, it did not show a statistically significant effect on obesity-relevant parameters such as eating behavior and body perception. Future studies should integrate VR systems into weight loss programs to investigate their potential as an additional tool for weight loss.

## Data Availability

The datasets used and/or analyzed during the current study are available from the corresponding author on reasonable request.

## Abbreviations

BMI: Body mass index
BSQ: Body Shape Questionnaire
CBT: Cognitive behavioral therapy
CI: Confidence interval
DEBQ: Dutch Eating Behavior Questionnaire
DSLR: Digital single-lens reflex
ECT: Experiential cognitive therapy
HMD: Head-mounted-display
MAIA: Multidimensional Assessment of Interoceptive Awareness
RCT: Randomized controlled trial
SBP: Standard behavior inpatient program
SD: Standard deviation
ViTraS: Virtual Reality Therapy by Stimulation of Modulated Body Perception
VR: Virtual reality
WHO: World Health Organization
